# The complete chloroplast genome of *Ocimum tenuiflorum* L. subtype Rama Tulsi and its phylogenetic analysis

**DOI:** 10.1080/23802359.2021.1944381

**Published:** 2021-07-05

**Authors:** Prakash Harini, Raju Balaji, Madasamy Parani

**Affiliations:** Center for DNA barcoding, Department of Genetic Engineering, SRM Institute of Science and Technology, Kattankulathur, India

**Keywords:** Complete chloroplast genome, Lamiaceae, *Ocimum tenuiflorum*, Rama Tulsi, phylogenetic analysis

## Abstract

*Ocimum tenuiflorum* L. subtype Rama Tulsi is an important aromatic perennial herb. It belongs to the family of Lamiaceae. In this study, the complete chloroplast genome sequence of *O. tenuiflorum* subtype Rama Tulsi was assembled and annotated using Illumina paired-end sequencing data. The length of the complete circular chloroplast genome was 151,722 bp. It comprises an inverted repeat (IR) region with a repeat length of 25,677 bp, a large single-copy (LSC) region of 82,781 bp, and a small single-copy (SSC) region of 17,587 bp. The GC content of complete chloroplast genome, LSC, SSC, IR regions is 37.9%, 36.0%, 31.8%, and 43.1%, respectively. The chloroplast genome contains 134 genes, including 88 protein-coding genes, 38 transfer RNA genes, and eight ribosomal RNA genes. Phylogenetic analysis with the complete chloroplast genomes of other related species revealed that the *O. tenuiflorum* L. subtype Rama Tulsi is fully resolved in a clade with other *Ocimum* species classified under the Lamiaceae family.

*Ocimum tenuiflorum* (L.) subtype Rama Tulsi is an important aromatic perennial herb distributed in the world's tropical and sub-tropical regions. It belongs to the Lamiaceae family. In the Ayurveda system of traditional medicine, Tulsi is commonly referred to as ‘Elixir of Life’ for its healing properties and its ability to cure various medical conditions (Jamshidi and Cohen 2017). *Ocimum tenuiflorum* has two botanically and phytochemically diverse subtypes that include Rama Tulsi with green leaves and Krishna Tulsi with purple leaves (Kothari et al. 2005). The leaf extract of Rama Tulsi was used for treating bronchitis, rheumatism, pyrexia (Nadkarni 2009). Other therapeutic uses of Rama Tulsi include treating epilepsy, asthma, cough, skin diseases, hematological diseases, wounds, to reduce tooth pain (Hebbar et al. 2004), and inflammations (Joshi et al. 2017). The roots and stems of Rama Tulsi were traditionally used to treat malaria and snake bites (Asolkar et al. 1992). *Ocimum tenuiflorum* leaf decoction can boost immunity against the novel coronavirus disease (COVID-19), possibly through the action of linalool, nevadensin, and 7-epi-sesquithujene (Khanal et al. 2020). In this study, we assembled and annotated the complete chloroplast genome sequence of *O. tenuiflorum* L. subtype Rama Tulsi for the first time using Illumina paired-end sequencing data to contribute to the genomic and genetic resource of this taxon.

The *O. tenuiflorum* L. subtype Rama Tulsi sample was collected from the campus of the Foundation for Revitalization of Local Health Traditions (FRLHT), Yelahanka, Bengaluru, India. (GPS coordinates: 13°07′24.5″N 77°32′52.3″E). Voucher specimen was deposited at the SRM Institute of Science and Technology Herbarium (https://www.srmist.edu.in/, Dr. Senthilkumar, senthilp3@srmist.edu.in) under the voucher number SRMH000143. The total genomic DNA was extracted from fresh leaves as described before (Poovitha et al. 2016). Nextera XT Library Prep Kit was used to construct a whole-genome DNA sequencing library. The DNA library (2 × 150 bp) was sequenced on the Illumina NovoSeq 6000 platform (Illumina, USA), and 1.01 Gb of paired-end sequencing data were generated. The chloroplast genome was assembled using NovoPlasty v4.3.1 (k-mer 33) (Dierckxsens et al. 2017) with *O. gratissimum* L. (Balaji et al. 2021) as a reference seed sequence. The annotation of the assembled chloroplast genome was performed using GeSeq (Tillich et al. 2017), taking the chloroplast genomes of *O. basilicum* (NC_035143.1) and *O. tenuiflorum* (NC_043873.1) as reference sequences. The predicted tRNAs were annotated by tRNAscan-SE 2.0 (Lowe and Chan 2016).

The complete chloroplast genome of Rama Tulsi is 151,722 bp in length and has a typical quadripartite construction that contains an inverted repeat (IR) region with a repeat length of 25,677 bp, flanked by a large single-copy (LSC) region of 82,781 bp and a small single-copy (SSC) region of 17,587 bp. The GC content of complete chloroplast genome, LSC, SSC, IR regions is 37.9%, 36.0%, 31.8%, and 43.1%, respectively. The complete chloroplast genome contains 134 genes that include 88 protein-coding genes, 38 transfer RNA (tRNA), and eight ribosomal RNA (rRNA). Introns are present in 16 annotated genes. Among them, nine protein-coding genes *(atpF, ndhA, ndhB, petB, petD, rpl2, rpl16, rps16,* and *rpoC1*) have single intron, and two protein coding genes (*ycf3* and *clpP*) have two introns. In addition, three tRNA genes (trnK-UUU, trnL-UAA, and trnV-UAC) have single intron and two tRNA genes have (trnE-UUC and trnA-UGC).

A neighbor-joining tree with 1000 bootstrap replicates was performed using MEGA version X (Kumar et al. 2018) from the alignments created by the MAFFT program (Katoh and Standley 2013). *Nicotiana tabacum* L. (Solanaceae) and *Arabidopsis thaliana* L. (Brassicaceae) were designated as outgroup taxa, and 20 published chloroplast genomes or the CDS of rbcL gene from the Lamiaceae were included as an ingroup. The phylogenetic analysis fully resolved the Rama Tulsi in a clade with other closely related *Ocimum* species (Figure 1). This complete chloroplast genome of *O. tenuiflorum* L. subtype Rama Tulsi can be subsequently used for species delimitation, phylogenetic analysis, DNA barcoding, and chloroplast genetic engineering studies of genus *Ocimum* and Lamiaceae family.

**Figure 1.**
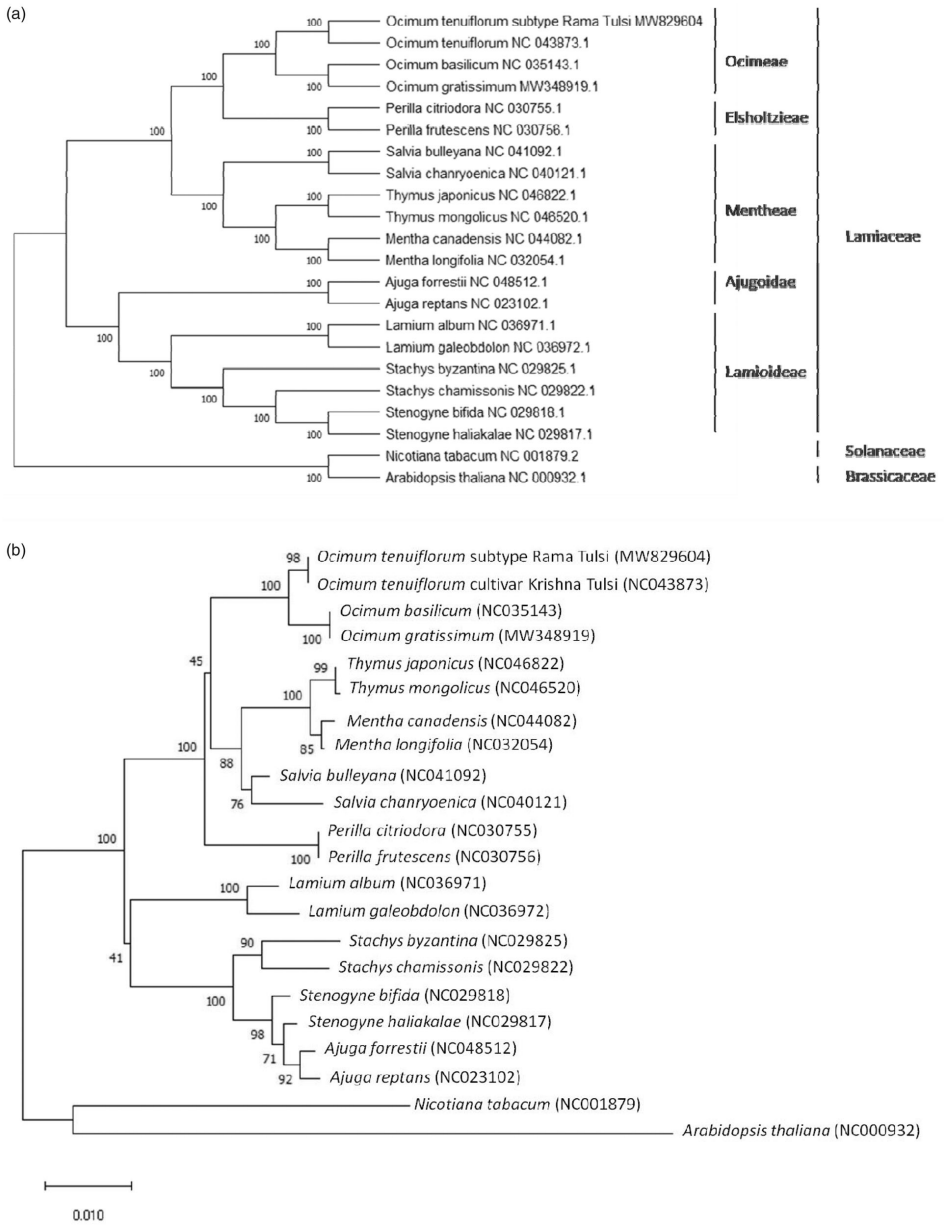
Neighbor-Joining (NJ) trees based on 22 complete chloroplast genomes (a) and CDS of *rbcL* (b) including *Nicotiana tabacum* L. and *Arabidopsis thaliana* L. as outgroup. The sequences were aligned using MAFFT online version (https://mafft.cbrc.jp/alignment/server/) and subjected to generating NJ phylogenetic tree using MEGA version X (Kumar et al. 2018). The bootstrap support values (>50%) from 1000 replicates are indicated in the nodes.

## Data Availability

The genome sequence data that support the findings of this study are openly available in GenBank at https://www.ncbi.nlm.nih.gov/genome under the accession number MW829604 (https://www.ncbi.nlm.nih.gov/nuccore/MW829604.1/). The associated NGS sequencing data files are available from the BioProject, SRA, and Bio-Sample ID under the accession numbers PRJNA718132, SRR14090688, and SAMN18523089, respectively.
